# Pancreatic islet organoids-on-a-chip: how far have we gone?

**DOI:** 10.1186/s12951-022-01518-2

**Published:** 2022-06-28

**Authors:** Jiaxiang Yin, Hao Meng, Jingfang Lin, Wei Ji, Tao Xu, Huisheng Liu

**Affiliations:** 1 Bioland Laboratory, Guangzhou, Guangdong China; 2 Guangzhou Laboratory, Guangzhou, Guangdong China; 3 National Laboratory of Biomacromolecules, CAS Center for Excellence in Biomacromolecules, Institute of Biophysics, Chinese Academy of Sciences, Beijing, China; 4 School of Biomedical Engineering, Guangzhou Medical University, Guangzhou, China

**Keywords:** Islet, Organoids-on-a-chip, Organoid culture, Disease modeling, Drug screening

## Abstract

Diabetes mellitus (DM) is a disease caused by dysfunction or disruption of pancreatic islets. The advent and development of microfluidic organoids-on-a-chip platforms have facilitated reproduce of complex and dynamic environment for tissue or organ development and complex disease processes. For the research and treatment of DM, the platforms have been widely used to investigate the physiology and pathophysiology of islets. In this review, we first highlight how pancreatic islet organoids-on-a-chip have improved the reproducibility of stem cell differentiation and organoid culture. We further discuss the efficiency of microfluidics in the functional evaluation of pancreatic islet organoids, such as single-islet-sensitivity detection, long-term real-time monitoring, and automatic glucose adjustment to provide relevant stimulation. Then, we present the applications of islet-on-a-chip technology in disease modeling, drug screening and cell replacement therapy. Finally, we summarize the development and challenges of islet-on-a-chip and discuss the prospects of future research.

**Graphical Abstract:**

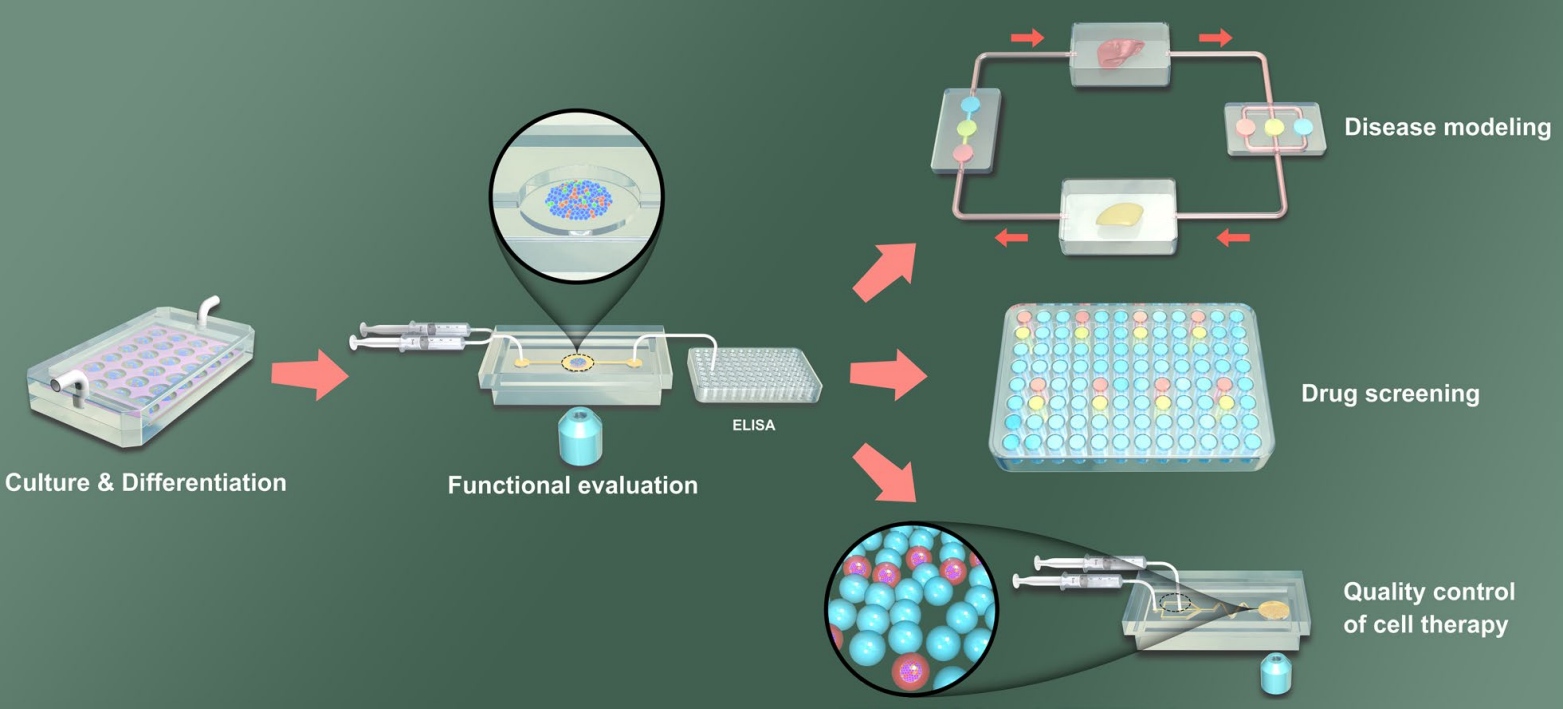

## Introduction

Diabetes mellitus has become a public health crisis in all countries worldwide. In 2019, the International Diabetes Federation (IDF) reported that the number of adults (20–79 years old) living with DM was approximately 463 million, equivalent to 9.3% of the total population of the world in this age group. By 2030 and 2045, the number of adult diabetic patients will increase to 578 million and 700 million, respectively. The islets of Langerhans release insulin directly into the blood circulation to maintain glucose homeostasis based on the changes in glucose levels, and DM occurs when this regulatory system is permanently disrupted [1].

Currently, most of the in vitro cell studies rely on 2D or suspension culture that fail to simulate the cell growth environment in vivo. For the past few years, considerable progress has been made in exploiting self-organizing properties of mammalian cells to produce organotypic multicellular structures called organoids [2–4]. Organoids are mini-tissues that are structurally and functionally similar to target organs that constructed by 3D culture technology. Besides, the connection between cells is more complex than simple physical contact, there exists mutual biological communication, influence, induction, feedback and cooperation to form micro-organs or tissues with specific functions. Therefore, organoids have great potency to simulate the specific structure or function of organs or tissues in vitro, which is unmatched by traditional 2D culture methods [5–7]. Due to the wide application prospects of organoids in the field of life science and medical research, they were rated as the technology of the year in 2017. However, the conventional 3D culture technology with random configuration makes it hard to precisely control organoid development and local environment. Furthermore, the existing 3D organoid culture methods cannot provide a complex dynamic microenvironment similar to in vivo for organ development [8]. By integrating organoids with microfluidic systems, organoids-on-a-chip can not only simulate the physiological environment of organ development, but also be applied in the simulation of complex disease processes [9]. By combining functional tissues and organs with chips, these systems enable analysis of the biochemical, metabolic and genetic activities of alive cells under high-resolution and real-time imaging. Moreover, this system is a powerful tool to advance research on development and physiology of organ, disease modeling and etiology, and drug screening.

**Fig. 1:**
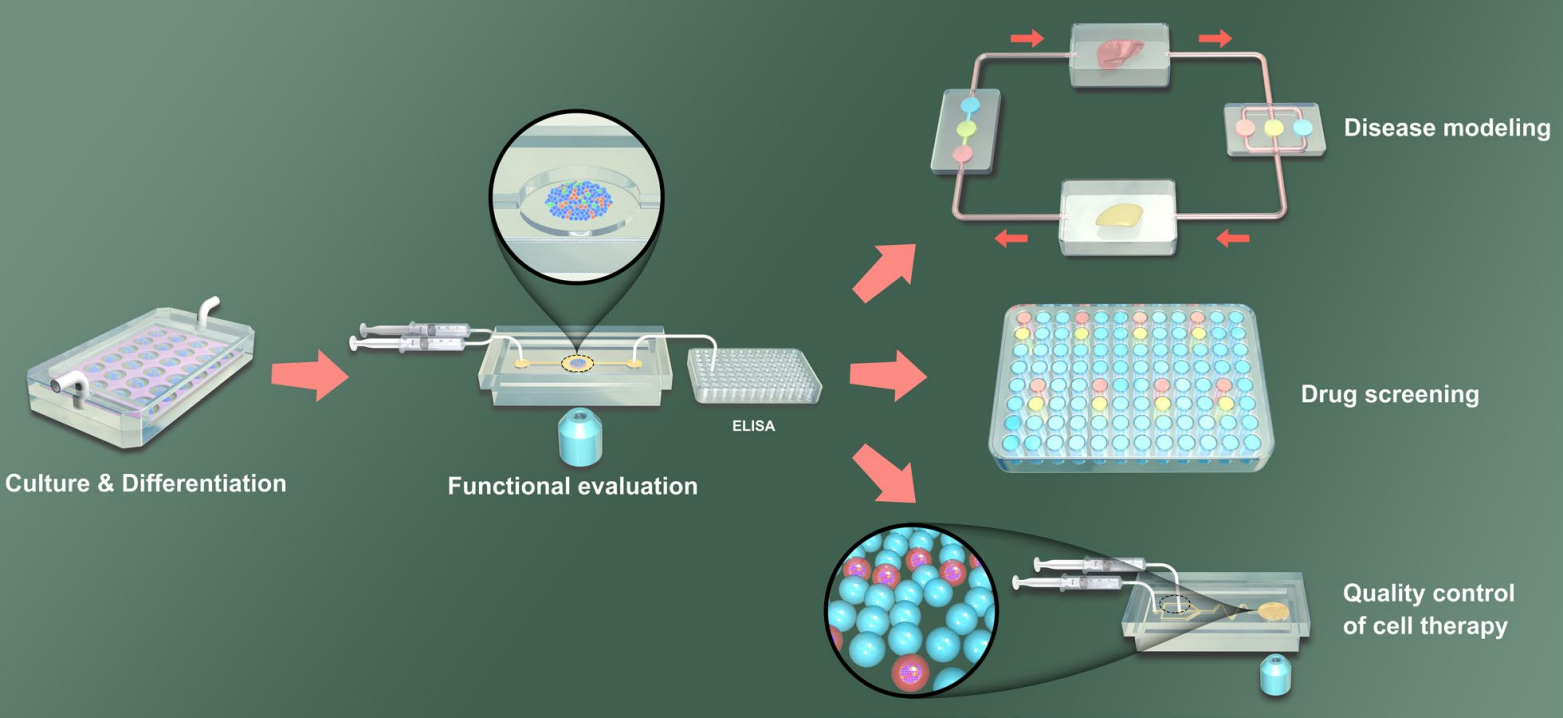
The application of organoids-on-a-chip platform in pancreatic islet research. Microfluidic platforms enable reproducibility of islet organoid culture and differentiation. Besides, these systems enable efficient functional evaluation of pancreatic islet organoids, such as single-islet-sensitivity detection, long-term real-time monitoring, and automatic glucose adjustment to provide relevant stimulation. Furthermore, islet organoids-on-a-chip platform can be applied in disease modeling, drug screening and the quantity control of cell therapy for diabetes mellitus

Here, we introduce a comprehensive review of the development, application, challenge and prospect of islet-on-a-chip platforms (Fig. 1). We first introduce recent advances in islet-on-a-chip, including microfluidic perfusion systems for stem cell differentiation, islet culture and functional evaluation. Then, we present the applications of organoids-on-a-chip in disease modeling, drug screening and the quantity control of cell therapy for DM. Finally, we summarize the challenges and future prospects of pancreatic islet organoids-on-a-chip.

## Chip for islet organoids culture and differentiation

Although traditional 2D or 3D cell culture methods have been widely applied in biomedical research in the past century [10, 11], they have limitations in the recapitulation of dynamic physiological 3D microenvironments for organoid generation. Furthermore, cells in such systems are not typically exposed to normal mechanical signals, containing tension, compression and fluid shear stress [12]. Especially, the development and formation of islets of Langerhans is strictly regulated by complex and dynamic factors, mainly including flow of blood, transcription factors, and interaction between cells, to generate functionally mature islets to maintain blood glucose homeostasis [13]. Advances in microfluidic-based cell culture systems have the ability to establish cellular microenvironments that simulate many important in vivo characteristics through precise control of the flow of fluid, biochemical signals, and interaction between cells. Moreover, many studies have generated islet-like organoids by integrating microfluidic-based cell culture systems with self-organizing properties of stem cells or mammalian cells [14–19].

### Encapsulation of human islet organoids in hydrogel scaffolds and cultured in a static culture system

**Fig. 2:**
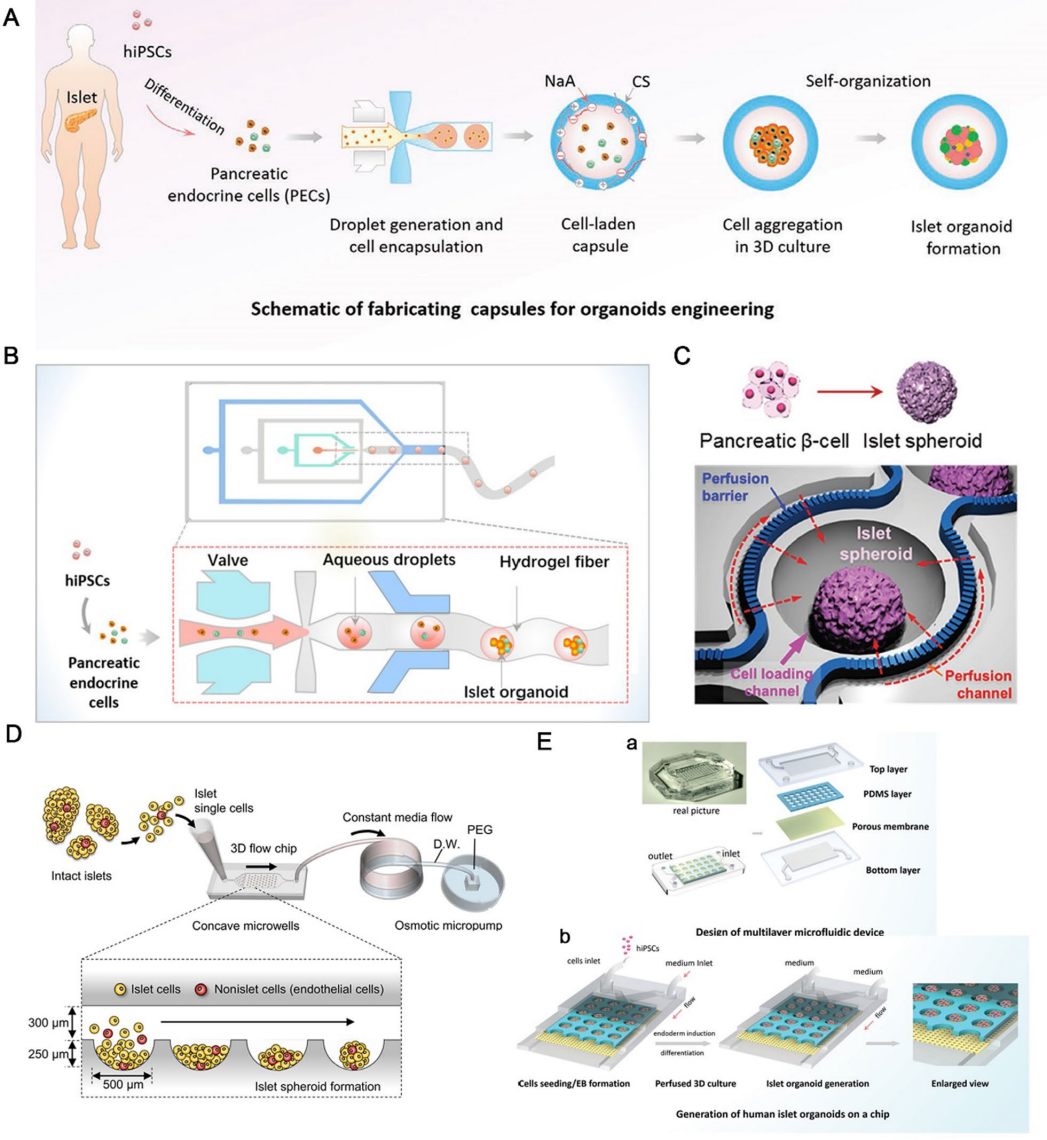
Microfluidic systems for islet organoid culture and formation. **A** An all-in-water droplet microfluidic platform for fabrication hybrid hydrogel capsules enabling islet organoid culture and formation. Reproduced with permission from Ref. [20]. **B** An aqueous-droplet-filled hydrogel fibers fabricated system for islet organoid culture and formation. Reproduced with permission from Ref. [21]. **C** The microphysiological analysis platform that permits generate islet spheroids under a perfusion flow network. Reproduced with permission from Ref. [22]. **D** The in vivo-mimicking chip allows reduce shear damage to cells, and generates functional islet spheroids and maintains long-term islet function. Reproduced with permission from Ref. [23]. **E** The multilayer microfluidic perfusion system permits in situ monitoring of islet organoid culture and develop. Reproduced with permission from Ref. [24]

To stably generate islet organoids in large quantities, Qin et al. [20] fabricated an all-in-water droplet microfluidic device for 3D culture of human-induced pluripotent stem cells (hiPSCs) to differentiate functional islet organoids with uniform size. Figure 2 A shows the whole process of this system utilized for hiPSC-derived islet organoid engineering. The platform consists of multiphase fluid inlets (pump core flow, middle flow and shell flow into the corresponding microchannels), droplet generation units (form drop templates), and capsule fabrication units (form hybrid capsules). This platform encapsulated pancreatic endocrine cells derived from hiPSCs into hybrid hydrogel capsules to obtain a continuous generation of 3D organoids composed of α and β cells through self-organization. Islet organoids generated using this platform highly express genes and proteins of pancreatic hormone, and show islet-specific function of glucose-stimulated insulin secretion (GSIS). By changing materials pumped into the droplet microfluidic platform, the same group fabricated aqueous-droplet-filled hydrogel fibers (ADHFs) in one step (Fig. 2B) [21]. The ADHFs produced by this platform have unique morphology and tunable configuration. After digesting pancreatic endocrine cells, the droplet microfluidic platform was used to encapsulate cells into hydrogels, and generated ADHFs with cells were collected using a rotary spool and rapidly shifted to a culture plate. Functionalized islet organoids were generated in situ and these organoids showed high viability and sensitive GSIS function.

### Dynamic perfusion culture of islet organoids-on-a-chip

Although the droplet microfluidic platform allows large-scale 3D culture and generation of uniform and functional islet organoids, the static culture environment limited to reproduce the dynamic physiological microenvironment of islet development in vivo, such as the continuous exchange of oxygen, nutrients, and metabolites through perfusion. Lee et al. [22] fabricated a microphysiological analysis platform (MAP) that can generate islet spheroids under a perfusion flow network (Fig. 2 C). The MAP allows continuous dynamic perfusion, uniform 3D spheroid formation, massive morphological phenotyping and gene expression profiling. This platform mainly consists of 3 units: medium storage area (length, width and height are 15, 15 and 7 mm, respectively), cell access channel (width and height are 200 μm and 30 μm, respectively), and 3D cell culture area (This area consists of two parts, one is a single islet spheroid culture chamber with a diameter and height of 800 μm and 300 μm, and the other is an endothelial-like cell entry channel with a width and height of 100 μm and 30 μm). In particular, there exists a periodic narrow slit structure (with a width of 2 μm and an interval of 20 μm) on the upper of the perfusion channel, which allows medium exchange in islet spheroid culture region. The whole size of the fully assembled MAP device is 70 × 22 × 9 mm, including 110 parallel spheroid culture arrays. The microfluidic-based MAP enables the generation of islet spheroids without scaffold and accurate control of blood glucose and lipidemia under a perfusion flow network. Based on this platform, the authors confirmed that cellular apoptosis in islet spheroids under hyperglycemia and hyperlipidemia mainly depends on the caspase-mediated pathway induced by reactive oxygen species.

Microfluidic perfusion systems are able to mimic in vivo physiological environment to improve the culture microenvironment of cells; however, the effects of fluid flow on the viability and function of islets are usually ignored. By optimizing flow conditions, Lee et al. [23] established a microfluidic perfusion chip that mimics interstitial flow (Fig. 2D). The chip reduces shear damage to cells, thereby generating functional islet spheroids and maintaining long-term islet function. The microfluidic chip contains two parts: PDMS-based concave microwells and an osmotic pump. The width and height of the concave microwells are 500 μm and 250 μm, and there are 50 microwells on a single chip. The osmotic pump that connects to the outlet of the concave microwells area is driven by the concentration difference between pure water and polyethylene glycol (PEG) (0.05 or 0.20 M) to provide an interstitial flow. The chip maintains the morphology and function of islet spheroids for up to a month due to the optimized flow conditions that allow the localization of secreted soluble factors close to the islets and promote intra-islet diffusion-mediated paracrine interactions. Furthermore, the functional islet spheroids with good viability and uniform size are generated using this chip and exhibit high drug sensitivity. Therefore, this chip has great potential for application in islet preprocessing before cell transplantation therapy and as a reliable in vitro model for drug screening of DM.

To achieve in situ monitoring of islet organoid culture and develop on a microfluidic perfusion chip, Qin et al. [24] developed a multilayer microfluidic system. The multilayer microfluidic perfusion system (Fig. 2E) contains 4 layers. The upper and lower layers are made with a chamber (the length, width and height are 10, 5 and 1 mm, respectively) with one inlet and one outlet for culture medium or cells input and output. The system permits the aggregation of embryoid bodies, differentiation of islets and formation of organoids of uniform size under continuous perfusion 3D culture. The islet organoids generated in this system contain α and β cells, and show good growth and viability. Compared to static culture, these islet organoids show increased genes and proteins expression of β cell, such as PDX1, NKX6.1, insulin and C-peptide. Furthermore, these organoids show enhanced GSIS and higher Ca^2+^ influx, suggesting that the biomimetic perfusion system contributes to the differentiation of hiPSCs and the functional maturation of pancreatic organoids.

## Chip for evaluation of islet organoid function

Insulin release function of cadaveric or stem cell-derived islet is routinely evaluated before transplantation to test the therapeutic potency [25–28]. Currently, the general method for evaluating pancreatic islet is the detection of insulin secretion, which includes the tedious manual division of islets into aliquots, tedious liquid-handling, and enzyme-linked immunosorbent assays (ELISAs), which are time consuming. Microfluidic technology is a promising method to evaluate pancreatic islet function based on its unbeatable advantages of automation and integration, portability, high throughput, low reagent/sample consumption, and pollution prevention [29, 30]. Currently, various microfluidic systems have been established to evaluate islet organoid function, including the evaluation of insulin secretion [31], Ca^2+^ flux [32, 33], fatty acid oxidation [34], oxygen consumption [35, 36], cell membrane capacitance and cytoplasmic conductivity [37]. These methods have many advantages over traditional methods, such as single-islet-sensitivity detection, long-term real-time monitoring and automatic glucose adjustment to provide relevant stimulation [38–41].

### On-chip insulin secretion collection and off-chip immunoassay

**Fig. 3:**
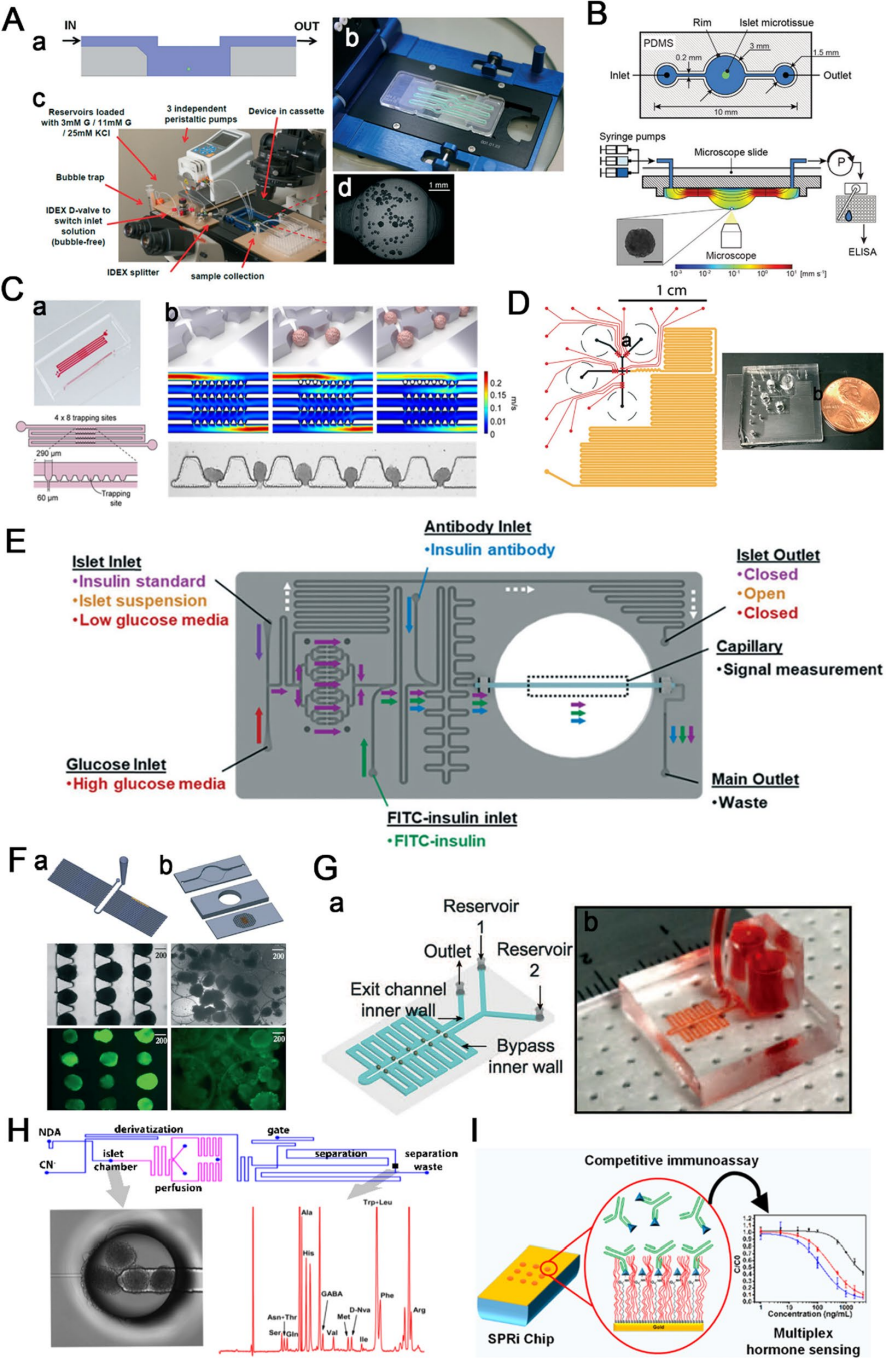
Microfluidic systems for functional measurement of pancreatic islet. **A** The 3-well fluidic platform (FP-3 W) with optimized convective fluid transport allows the dynamic glucose-stimulated insulin secretion evaluation. Reproduced with permission from Ref. [42]. **B** The microfluidic hanging-drop-based perfusion platform for evaluating dynamic glucose-stimulated insulin secretion of single islet. Reproduced with permission from Ref. [44]. **C** The microfluidic system integrated with a Raman microspectroscope for marker-independent evaluation of pancreatic islet. Reproduced with permission from Ref. [45]. **D** The fully automated droplet generation and analysis device for sampling and quantification of islet secretion. Reproduced with permission from Ref. [46]. **E** The microfluidic-based islet-on-a-chip that enables automate islet loading, stimulation and insulin quantification. Reproduced with permission from Ref. [47]. **F** The microfluidic array enables live-cells muti-parameters imaging. Reproduced with permission from Ref. [48]. **G** The chip with cup-shaped nozzles for limiting shear-induced damage and imaging the Ca^2+^ responses of pancreatic islet. Reproduced with permission from Ref. [49]. **H** The microfluidic platform for online monitoring of amino acid secretion from islet in response to glucose stimulation. Reproduced with permission from Ref. [50]. **I** The surface plasmon resonance imaging chip (SPRi Chip) for simultaneous quantification of 3 islet secretions. Reproduced with permission from Ref. [51]

Combining computational fluid dynamics model, Agarwal et al. [42] developed a PDMS-free 3-well fluidic platform (FP-3 W) with optimized convective fluid transport for functional assessment of pancreatic islet (Fig. 3A). FP-3 W is manufactured using rapid prototyping technology and made of bioinert acrylic plastic material. The width (diameter) and height of the well are 4 and 1.5 mm. The size and structure of the well are designed to mimic 96-well plate to restrict response delays caused by excessive dead volumes. Additionally, this design also facilitates pancreatic islet loading and retrieval. After hand-picked rodent or human pancreatic islets (between 10 and 60 each well) are loaded into the wells, the platform allows the dynamic testing of pancreatic islet for GSIS and simultaneous live-cell imaging. Experimental results demonstrate that the platform with geometrical features, designed based on first principles-based computational modeling, adequately perfuse the loaded pancreatic islets and permit for timely collection of the samples. In addition, dynamic insulin secretion of pancreatic islet (flow rate, 50 µL min^− 1^; sampling rate, 2 min per well) could be detected with sensitivity of as few as 20 islets per well. By integrating the FP-3 W with 3D alginate hydrogel, Stabler et al. [43] developed a new system that allows dynamic culture and continuous in situ multiparametric evaluation of rodent or human pancreatic islet. Under continuous, high-throughout, and non-invasive evaluation, this system can maintain pancreatic islet viability and function for more than ten days, in sharp contrast to the dramatic decline observed overnight under static conditions. Using this system, the same set of pancreatic islets were exposed to continuous, dynamic fluid system over extended culture periods, and for the first time the dynamic GSIS, in situ tracking of activity and calcium signaling were investigated simultaneously.

To reduce sample variability and improve temporal resolution when testing insulin secretion, Frey et al. [44] developed an analytical platform based on microfluidic hanging drop perfusion. The platform with rapid stimulus solution exchange further reduced sample dilution rates, reduced analyte dispersion and increased sampling intervals. The layout and dimension of the perfusion system are shown in top and cross-sectional view (Fig. 3B). The primary islets are dispersed by enzymatic dissociation, and the cell dispersion is re-aggregation in controlled scaffold-free hanging-drop to generate standardized pancreatic islet microtissues (approximately 150 μm). After loading islet microtissues, a syringe pump is set at the inlet to pump medium at 15 µL min^− 1^, and a peristaltic pump is set at the outlet to withdraw medium samples into a 384-well plate at 15 µL min^− 1^. Finally, the dynamic GSIS evaluation of islet microtissue is performed off-line using ELISAs. The platform was designed to set different parameters flexibly to characterize insulin release from individual pancreatic islet microtissues.

### One-step on-chip insulin secretion and assessment

Although common off-chip immunoassays can monitor the concentration of insulin secreted from pancreatic islet, on-chip assessment can more accurately evaluate the function of pancreatic islet in real time. To track GSIS in real-time on-chip, Loskill et al. [45] established a microfluidic system integrated with a Raman microspectroscope device for functional assessment of human pseudoislet. The chip contains a 4 × 8 trapping site array, and each trapping site can capture a single islet (Fig. 3 C). Human pseudoislets that derived from immortalized cell line EndoC-βH3 are introduced to the chip automatically at the inlet by hydrostatic flow and captured in the trapping sites without altering viability. After the trapping sites are filled during loading, extra islets flow out from the outlet along with the medium. The results demonstrated that Raman microspectroscopy can in situ track the islet responsiveness to glucose and visualize the different molecular structures of nuclei, lipids and mitochondria. Furthermore, in-depth spectral analysis showed that glucose stimulation is correlated with mitochondrial activity in islet cells, and lipid composition of insulin-secreting vesicles is changed simultaneously.

Using fluorescent labeling and real-time microscopic imaging, Easley et al. [46] developed a fully automatic droplet formation and analysis system for the on-chip assessment of pancreatic islet (Fig. 3D). This automated device generates droplets by the alternating inputs of aqueous and oil phases and can precisely control droplet size, oil spacing, and the ratio of sample and reference droplets. Based on homogeneous immunoassay, the chip can quantify insulin secretion from single pancreatic islets directly from droplets using the optical readout. Using this system, the quantitative measurement of GSIS can be completed with a temporal resolution of 15 s and rapid insulin oscillations reflect the changes of intracellular calcium signals. In addition, the sensitivity of the system is very high and the detection limit is as low as 10 amol per droplet.

### Scalable manufacturing of chip for insulin assessment

Although microfluidic chip has many advantages in evaluating the function of the islet of Langerhans, traditional methods are still widely used due to the complexity of serial real-time dynamic detection on-chip and the difficulty of large-scale manufacturing of microfluidic chips. By integrating scalable manufacturing materials and based-fluorescence anisotropy on-chip immunoassay, Parker et al. [47] fabricated an islet-on-a-chip platform with automatic islet loading, stimulation, and insulin quantification (Fig. 3E). The chip was fabricated from polycarbonate material using a computer numerical control mill. The chip has 4 main parts: the islet and glucose inlet (for introducing islets and solutions), the culture area (containing 16 traps set for islet trapping), FITC-insulin and antibody inlets and mixing channels (for FITC-insulin and antibody pumping and mixing), and a glass capillary (for insulin quantification). Islets that suspended in the insulin-free medium are pumped through the islet inlet and captured in the islet traps. At this point, the islet outlet is opened to permit excessive islets to flow out. The islet outlet is closed after the traps are filled with islets. Concentration-dependent dynamic glucose stimulation of trapped islets is obtained by simultaneously pumping different flow ratios and different concentrations of glucose at the inlet. In the process of glucose stimulation, FITC-labeled insulin and insulin antibody are introduced and mixed with insulin secreted from the trapped islets. Finally, concentration of insulin secreted from the trapped islets is instantly and continuously monitored by fluorescence anisotropy. The thermoplastic microfluidic chip enables full automation from islet loading to insulin quantification. Most importantly, the chip could be large-scale manufacturing, which will accelerate quantification of massive islets on diabetes treatment.

### Other functional evaluations of islets

Regarding the functional evaluation of pancreatic islet, while many researchers focus on insulin secretion, others are interested in studying the other features of islet, such as physiological or pathophysiological behavior [36, 48], glucose-stimulated Ca^2+^ response [49], the role of amino acids in regulating hormone release [50], and the paracrine interaction in cells of islet [51].

To study the physiological changes of single islets in health and disease, Wang et al. [48] fabricated a microfluidic array for live-cells multi-parameters imaging (Fig. 3F). The array contains 300 trapping sites. The U-cup shaped pocket trap site has a diameter and depth of 250 and 275 μm, respectively, and the size of the pocket gradually decreases from 250 μm to 45 μm from top to bottom. There exists a cross-flow channel overhead the pocket for continuous perfusion of solution. The design of the array achieves 95% capture efficiency of pancreatic islets, and provides precise control of flow dynamics, speeds up solution delivery, shortens stimulus response time, and improves the sensitivity and accuracy of the assay. Importantly, the device enables the monitoring of a lot of single islets by live-cell multiparametric imaging, which is very useful for evaluating islet viability and function, and screening antidiabetic drugs.

When β-cells are stimulated by high concentrations of glucose, they respond by membrane depolarization [52]. When cells are stressed or damaged, the glucose metabolic and Ca^2+^ responses of pancreatic islet are lost. To prevent shear-induced damage to cells in the perfusion system, Rocheleau et al. [49] developed a platform to capture islets in continuous cup-shaped nozzles for the assessment of islet function (Fig. 3G). The platform consists of a 125 μm high channel with multiple cup-shaped nozzles (300 μm wide and rounded to 50 μm) sequentially connected through bypass channels. This design allows islets to flow into cup-shaped nozzle and be captured by the openings (50 μm wide). When the opening of cup-shaped nozzle is blocked by an islet, continuous medium flow through the bypass channel. Meanwhile, the flow rate around the perimeter of islets is limited while the flow of medium through the tissue is enhanced. Importantly, when the islets are treated with this platform, the morphology of endothelial cells is enhanced and the glucose-stimulated Ca^2+^ is unaffected.

It is hypothesized that amino acids released from islet or intrapancreatic neurons cells could regulate secretion of hormones. In contrary to the well-known mechanism of GSIS, the role of amino acids in regulating islet hormone release is still unclear. To investigate the role of amino acids in autocrine and paracrine of islet physiology, Roper et al. [50] fabricated a microfluidic platform to perform online monitoring of amino acid secretion profiles in response to glucose stimulation (Fig. 3 H). This device consists of an islet chamber (to perfuse stimulants) with a diameter of 600 μm and an amino acid quantification system integrated on a single microchip. The device is equipped with a voltage of -15 kV for the rapid, high-efficiency separation of amino acids. By optimizing the constituents of derivatization and separation solutions, islets are prevented from settling into the channel, allowing serial monitoring of insulin secretion for more than two hours. Using the device, 10 amino acids are separated from a mixture of 18 with a detection limitation ranging from 1 to 20 nM. The secretion rates of 9 kinds of amino acids secreted from islets are obtained by the gravity-driven perfusion device pumping 3 mM and 20 mM glucose to stimulate islets. Therefore, this device can be used to track amino acid secretion from islets and evaluate the function of islet.

While researchers have developed a number of microfluidic chips to monitor hormone secretion from pancreatic islet, none of them could monitor more than two hormones simultaneously. Hence, surface plasmon resonance imaging (SPRi) is an efficient tool for research the interaction of surface biomolecules, enabling high-content and multiplexed biomolecule quantification. By properly implementing hormones on a sensing surface, Tabrizian et al. [51] established a SPRi-based biosensor device for the simultaneous assessment of 3 islet secretions (Fig. 3I). The surface of this biosensor is composed of a hybrid self-assembled monolayer of CH_3_O-PEG-SH and 16-mercaptohexadecanoic acid, which exhibits excellent antifouling properties. The design enables the application of the biosensor for the detection of complex matrix. This biosensor has excellent antifouling performance and specificity, showing an ignorable response to bovine serum albumin (1 mg mL^− 1^) and a very low response to lysozyme. In addition, the detection limits of the biosensor for insulin, glucagon and somatostatin are 1 nM, 4 nM, and 246 nM under a multiplex mode and the total analysis time is within 21 min. This biosensor device provides the promise to monitor the paracrine effect of somatostatin on GSIS and to discover novel drugs for the treatment of DM.

## Chip for diabetes-related disease modeling

Much knowledge about DM and its treatment comes from animal models that are ‘humanized’ by implanting human primary lymphocytes or hematopoietic stem cells. However, there are many limitations in filling the translational gap between human research and clinical trials [53] due to the distinct differences between humans and animals in terms of islet structure [54], metabolism [55, 56], and general immune responses [57]. The emergence of organ-on-a-chip as an animal surrogate model plays a vital role in disease modeling due to its high reproducibility and ability to mimic the real human organ structure and function [58–60]. The pancreatic islets-on-a-chip platform, as a disease model, has facilitated recapitulating the state of pancreas-related diseases and studying the effects of drug treatment, such as cystic fibrosis (CF) and type 2 DM.

**Fig. 4:**
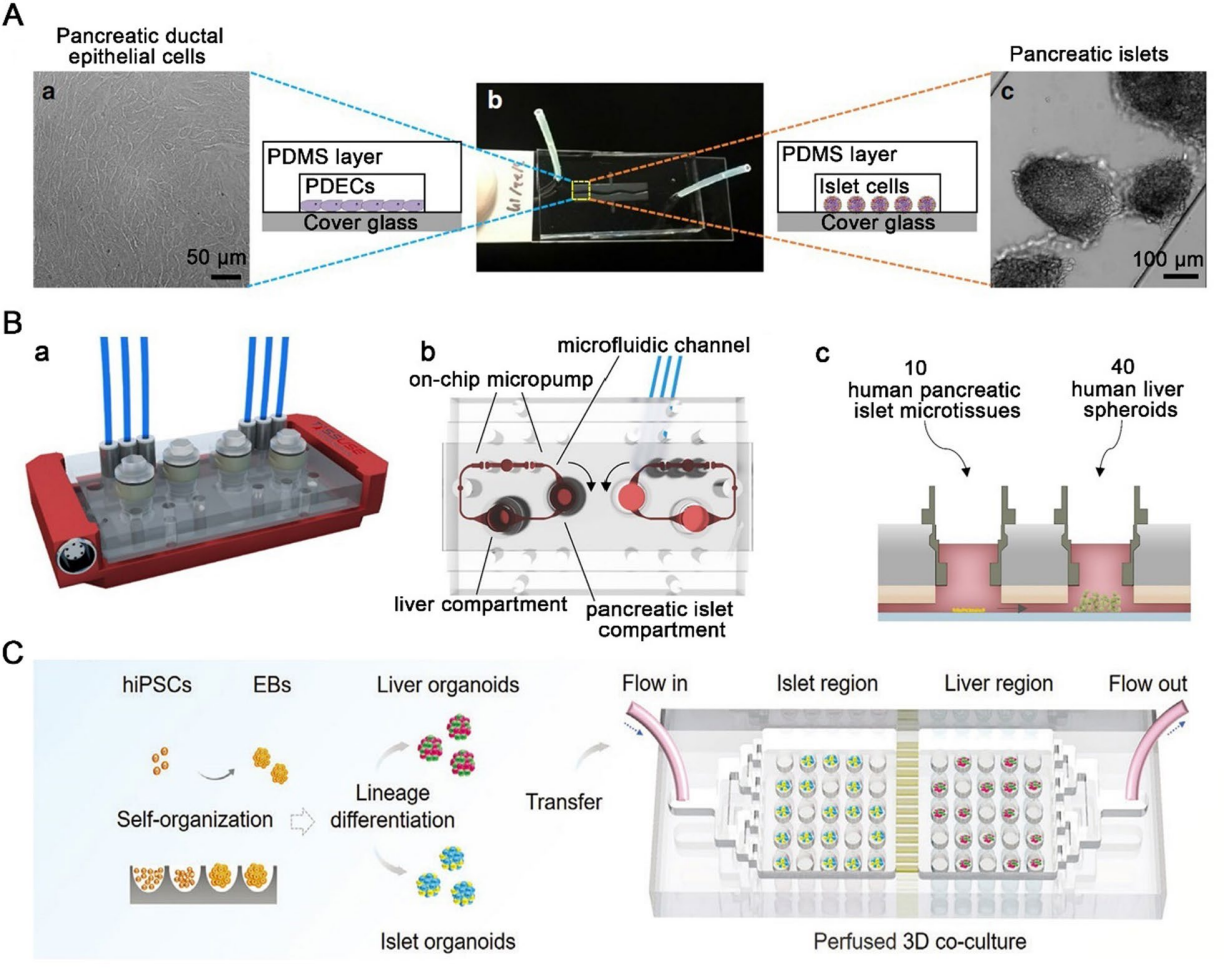
Pancreatic islets-on-a-chip for disease modeling. **A** Pancreas-on-a-chip co-culture system for researching cystic fibrosis-related diabetes. Reproduced with permission from Ref. [63]. **B** Islets and liver microtissues on-a-chip for studying type 2 diabetes mellitus. Reproduced with permission from Ref. [67]. **C** The microfluidic multiorganoid system for the interaction of liver and islet organoids in normal and disease states. Reproduced with permission from Ref. [68]

CF is a genetic disorder due to the dysfunction of cystic fibrosis transmembrane conductance regulator (CFTR). CF-related diabetes, the complication of CF, is a common fatal disease. People with CF have a 5% increased risk of diabetes per year, reaching 50% at the age of 40 [61]. As many as 2% of children, 19% of adolescents, and 50% of adults with CF are affected by CF-related diabetes [62]. One hypothesis is the insulin-producing islet locate adjacent to the pancreatic duct, and CF may impair intercellular signaling between islet cells and pancreatic duct epithelial cells. To further explore this possibility, Naren et al. [63] developed a co-culturing pancreas-on-a-chip platform. The customized platform consists of an upper layer with a cell culture chamber (for pancreatic ductal epithelial cells culture), a lower layer with a cell culture chamber (for pancreatic islet cells culture), and a porous membrane to separate the two chambers into a dual-chamber chip (Fig. 4 A). The results show that the absolute amount of insulin is reduced by 50% when the function CFTR is inhibited. Although this chip cannot fully mimic the physiological and pathological of in vivo pancreatic system, this finding reveals that the critical role of CFTR in maintaining endocrine function, which will offer important insight into the etiology of CF-related diabetes. In addition, this system can be used to study CF-related diabetes and glucose imbalance, measure the variability of blood glucose, determine the correlation between glucose levels and CFTR mutation type, and screen small-molecule drugs that improve glucose abnormalities in individuals with CF.

Recently clinical studies revealed that the number of type 2 DM patients associated with multiple organ complication is increasing [64–66]. Pancreas and liver are two important organs for maintaining blood glucose homeostasis and their microphysiological co-culture system provides an in vitro platform to simulate type 2 DM. To study the long-term interaction of physiology between pancreas and liver, Abdersson et al. [67] developed a two-organ chip system of islet and liver microtissues. The microphysiological two-organ chip contains two spatially separated chambers for culturing organ equivalents, and the two chambers are interconnected by a microfluidic channel (Fig. 4B). For glucose tolerance test, insulin released from islet spheroids under different glucose level showed that functional coupling contributes to the uptake of glucose by liver microtissues. Co-culture system of islet and liver microtissues maintained normal postprandial glucose concentrations (normal postprandial glucose concentration in humans: 5.9 ± 0.6 mM) in circulation, whereas the glucose concentrations in both mono-cultures (pancreatic islet-on-a-chip and liver-on-a-chip) remained at a higher glucose concentration (11 mM glucose in the medium:). These results showed that insulin secreted from islets stimulates liver microtissues to take up glucose, which cannot effectively consume glucose without insulin. Insulin secretion was reduced with decreasing glucose concentration, indicating that there is a functional feedback loop between the liver and islet spheroids. Therefore, by integrating a device that induce impaired glucose regulation can make this model an in vitro human type 2 DM platform. Although the two organs chip model allows the routine interrogation of cellular crosstalk, it fails to accurately control human organ-specific features and reproduce similar signaling feedback loops in vivo because of the mixed cell lines used.

To reproduce the human liver-pancreatic islet dual-organ feedback platform in normal and diseased states, Qin et al. [68] developed a new microfluidic multiorganoid system (Fig. 4 C). The circulatory perfusion system consists of two compartmentalized areas connected by multiple microchannels and enables the 3D co-culture of liver and islet microtissues for more than 30 days. Transcriptome analysis revealed that metabolism-related signaling pathways were activated in co-cultured organoids. What’s more, in glucose tolerance test, the multiorganoid system increased the sensitivity of GSIS of islet microtissues and enhanced glucose consumption by liver microtissues. Notably, both liver and islet microtissues exhibited defective function of mitochondrial and reduced glucose metabolism capacity under high-glucose culture, which were mitigated by metformin. Therefore, the multiorganoid-on-a-chip platform can reproduce the interaction between liver and islet microtissues under normal and disease conditions, providing an efficient system for in vitro studies of diabetes.

## Chip for drug screening

Currently, the in vitro evaluation of diabetes drugs is performed using mostly traditional two-dimensional cell culture methods. With increasingly in-depth research, two-dimensional cell culture technology has been continuously improved, but this single-layer, flat-plate in vitro static cell culture platform is difficult to reproduce the complex three-dimensional physiological environment in the body. Microfluidic chips have the ability to precisely control cell culture microenvironment and simulate the morphology and function of in vivo organ due to their flexible structural design and large-scale integration [69]. The rapid development of organ-on-a-chip makes it a powerful tool for tissue and organ simulation, which can be applied in drug screening [59, 60, 70].

**Fig. 5:**
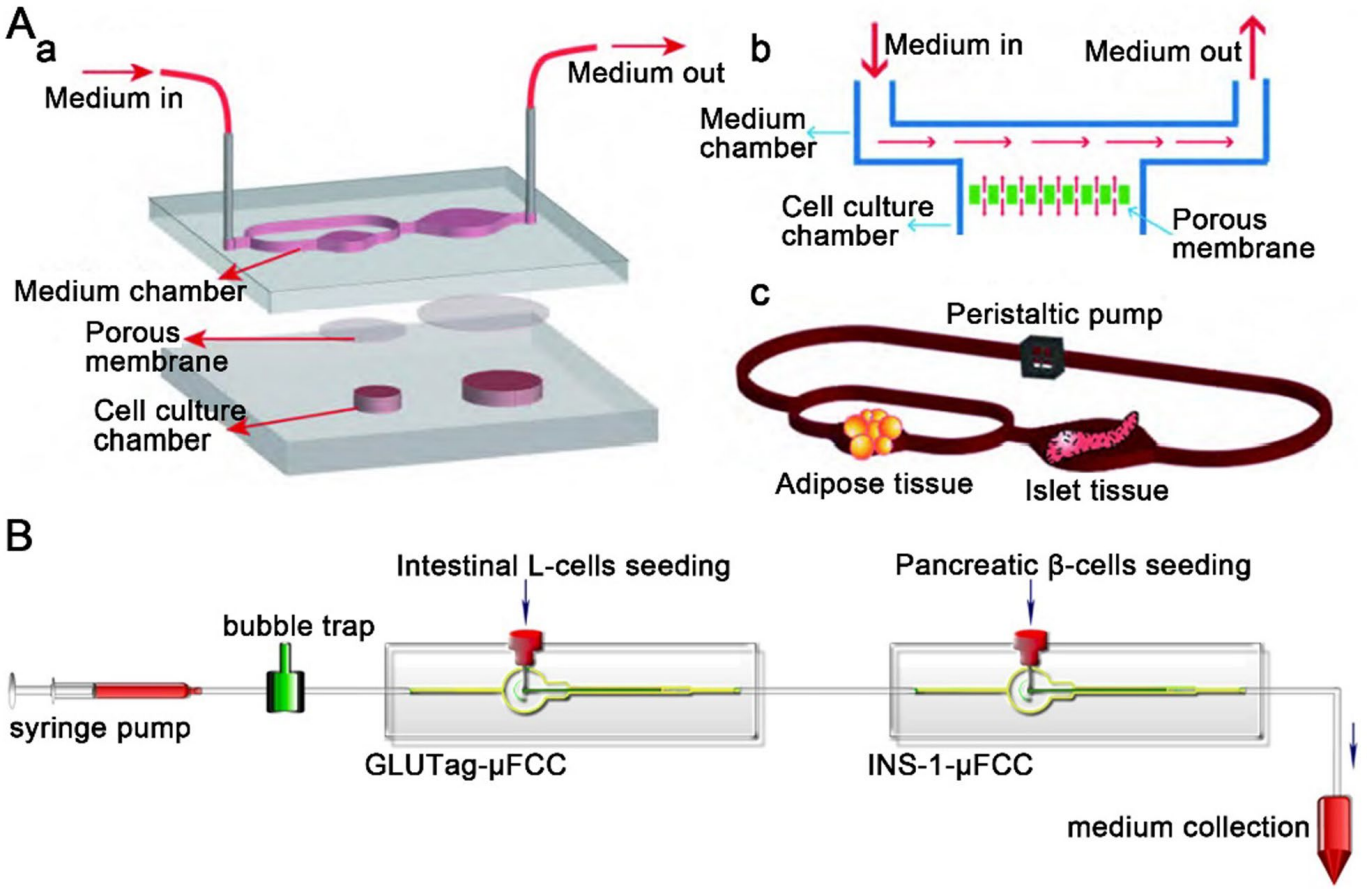
Pancreatic islets-on-a-chip for drug screening. **A** The fat and islet 3D double-organ chip for multiplexed assessment of type 2 diabetes drugs. R eproduced with permission from Ref. [71]. **B** Endocrine system on-a-chip to screen antidiabetic drugs. Reproduced with permission from Ref. [72]

As we recapitulate in the last section, the co-culture system of islets and other organs is an efficient disease modeling platform. In summary, most commonly used co-culture system to study the impact of organ crosstalk in type 2 DM including duct- and pancreatic islets-on-a-chip [63], liver- and pancreatic islets-on-a-chip [67, 68]. Fat is one of the most important tissues in the development of diabetes. To explore the interaction between fat and pancreatic islets in pathological conditions and to perform drug evaluation, Liu et al. [71] established a fat and pancreatic islet 3D double-organ chip. The chip consists of two chambers: one for adipose tissue culture (the diameter and height are 6 mm and 2 mm, respectively) and another for islet tissue culture (the diameter and height are 8 mm and 2 mm, respectively) (Fig. 5 A). There exists a polycarbonate membrane with a pore size of 10 μm between the channel and the tissue culture chamber to prevent the direct impact of fluid on the tissue. By analyzing the secretion of inflammatory factors such as adiponectin, interleukin 6 and interleukin 1β in adipocytes and pancreatic islet cells, insulin secretion ability, damage of islet cells, and inflammatory response can be analyzed in the system. The results show that lipopolysaccharide can cause inflammation and functional changes in pancreatic islet cells, and the presence of fat tissues can aggravate this response to a certain extent. Liraglutide reduces the inflammatory response of fat and pancreatic islet cells, which can reduce the stimulation of islet cells by lipopolysaccharide and adipose tissue to improve the function of islets. This fat and islet 3D double-organ chip can be applied to the multiorgan disease response caused by the interactions among tissues, exhibiting high potential as a key tool for drugs assessments for systemic metabolic diseases such as type 2 DM.

β-Cells in the pancreatic islet secrete insulin, while L-cells that stimulate insulin secretion are located in the small intestine. By measuring the effects on the production of glucagon-like peptide-1 (GLP-1) and insulin from GLUTag cell line and β-cell line under glucose-stimulated conditions, Park et al. [72] developed a co-culture small intestine- and pancreatic islet-on-a-chip for antidiabetic drugs screening. The chip contains an inlet (for medium inflow), a bubble trap district (for trapping bubbles), two chambers (for cell culture) and an outlet (for medium collection) (Fig. 5B). After three days of culture, both GLUTag and INS-1 cells formed aggregates and showed good viability (> 95%) and 3D cell morphology. The results showed that INS-1 cells co-cultured with GLUTag cells produced more insulin when stimulated with glucose. Furthermore, this small intestine- and pancreatic islet-on-a-chip showed faster saturation of insulin level at high glucose concentrations. Therefore, this system is a useful tool for the measurement of glucose-dependent dynamic changes in endocrine hormones and for the screening GLP-1 analogs and natural insulin for the treatment of DM.

## Chip for quality control of islets for cell therapy

Pancreas or islet transplantation to replace β-cells is currently the only long-term effective treatment for insulin-dependent DM patients. Despite good results after transplantation, pancreas transplantation is a major operation and may introduce serious complications. What’s more, the scarcity of donor organs for pancreas transplantation is a serious challenge. In contrast, islet transplantation to replace β-cells is a minimally invasive alternative with potentially wider indications owing to its low morbidity [73]. The rapid development of microfluidic chip technology and stem cell research has generated organoids-on-a-chip technology, which offers a sufficient source of islet organoids for islet transplantation. The quality control of islet organoids is vital to the success of the treatment, as the size of islet organoids as well as the maturity and morphology affect the therapeutic efficacy [74, 75]. Therefore, strict quality control of islets is an essential step before implantation into patients.

**Fig. 6:**
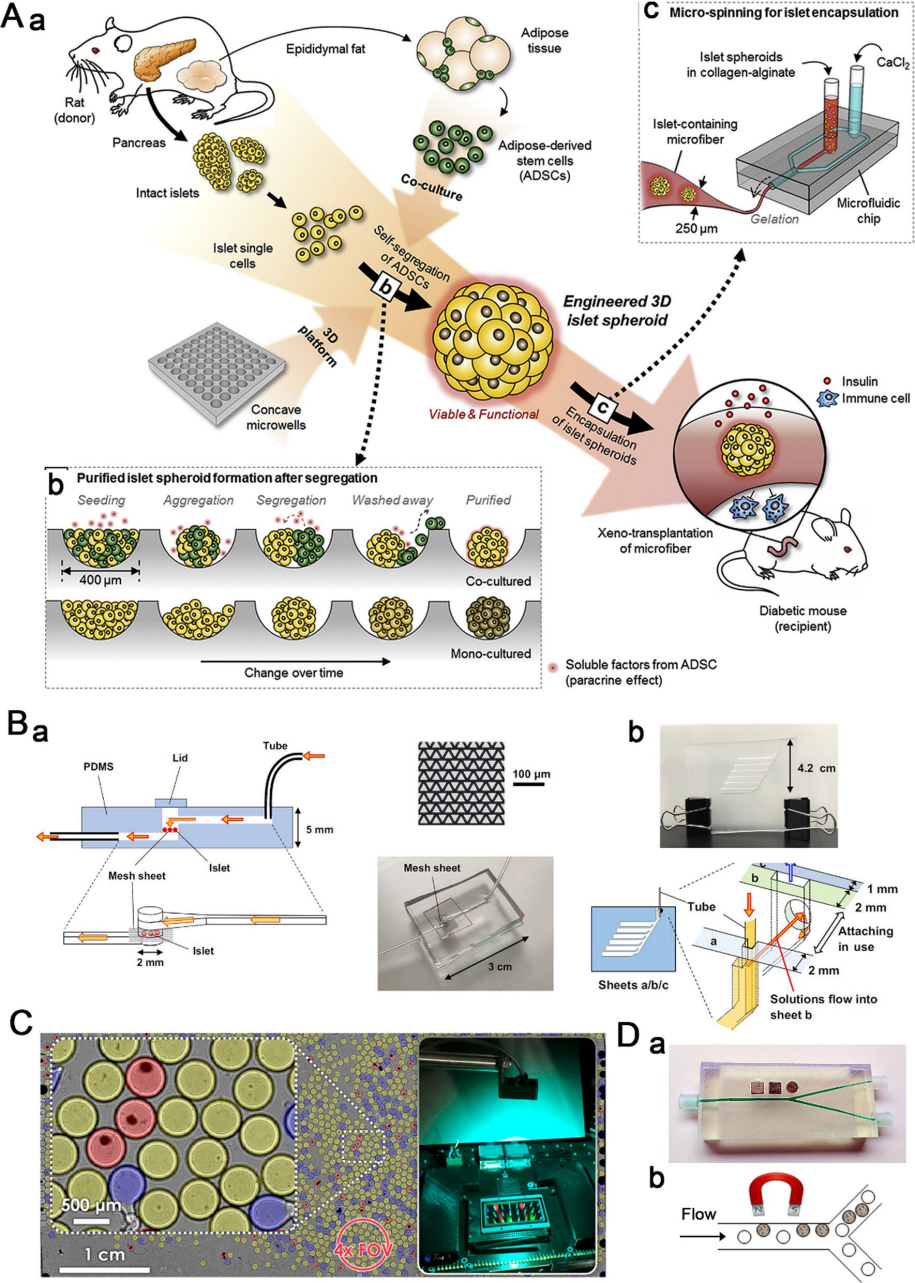
Microfluidic systems for quality control before islet transplantation. **A** The concave microwell chip for ensuring viable and function of islets for transplantation. Reproduced with permission from Ref. [76]. **B** The compact microfluidic system called g-STAR for evaluation of islet functionality for transplantation. Reproduced with permission from Ref. [77]. **C** The wide-field lens-free on-chip imaging system for high-content screening of islet microencapsulation for transplantation. Reproduced with permission from Ref. [82]. **D** The magnetic cell sorting system to purify microencapsulated islets for transplantation. Reproduced with permission from Ref. [83]

To create viable and functional islets for transplantation, Lee et al. [76] established a concave microwell chip for co-culturing single primary islet cells and adipose-derived stem cells (ADSCs) (Fig. 6 A). First, a mixture of single islet cells and adipose cells is seeded into a PDMS-based concave microwell array (the diameter and spacing are 400 and 500 μm, respectively) to obtain 3D co-cultured pancreatic islet spheroids. Co-cultured pancreatic islet spheroids form in a concave microwell array and are then encapsulated in microfibers on a PDMS-based microfluidic chip for xenogeneic transplantation. During islet formation in co-culture system, ADSCs segregate from islet cells, and purified islet microtissues are eventually generated. Compared to mono-cultured islet microtissues, the co-cultured islet microtissues demonstrate distinctly matured ultrastructural morphology, increased viability, and enhanced insulin secretion. Furthermore, xenotransplantation of these co-cultured islet spheroids into diseased mice showed longer maintenance of blood glucose levels with less islet mass than the transplantation of monocultured islet spheroids. Therefore, this method of culturing pancreatic islet spheroids can potentially provide reliable islets for clinical applications.

To efficiently assess islet functionality before transplantation, Iwata et al. [77] fabricated a compact fluidic device called g-STAR. The system contains two segments: the micromesh chip used as a fluidic device for pancreatic islets and the detection chip used to analyze the patterns of insulin secretion (Fig. 6B). Islets are loaded onto the mesh sheet, and medium pumped into the chip is able to reach the mesh sheet quickly, suggesting that this chip can be used for rapid GSIS analysis. The detection chip allows the sequential collection of sample solutions without mixing with fractionated solutions in a single chip. The system successfully analyzes the insulin secretion levels and patterns of the mouse islets, indicating its ability to easily and cost-effectively evaluate the quality of islets.

Islet transplantation has been used for the treatment of DM; however, the utility is limited even with long-term immunosuppression due to immune rejection of recipient [78]. To overcome this issue, islets were encapsulated with biocompatible semipermeable membrane to form capsules with diameter ranging from several hundred microns to over 1 mm [79–81]. The biocompatible membrane protects implanted islets from attack by immune cells without affecting the pass through of nutrients and insulin. However, the size, the morphology and the position of islets within the microencapsulation affect the therapeutic efficacy. Therefore, an effective platform for the quality control of microcapsules before implantation is essential. To replace the low throughput, high variation and labor-intensive process of the manual microscopic inspection of encapsulated islets, Ozcan et al. [82] developed an ultrahigh-content lens-free on-chip imaging system to rapidly screen alginate-encapsulated islets. The system integrates an optical setup with an ultrawide field of view and a custom-designed sample chamber (Fig. 6 C). The lens-free optical setup has a view field of 18.15 cm^2^, allowing it to image and analyze approximately 8000 islet microencapsulation in a single frame. Moreover, custom-written software for image reconstruction and processing provides users with detailed information on islet microencapsulation for transplantation, such as number, size and integrity of microencapsulation, and whether each microencapsulation contains islets. However, the system fails to identify the quality of encapsulated islets, which could reduce the islet graft volume and immune reaction after implantation.

To exclude the empty capsule from therapeutic graft in DM, Pedraz et al. [83] established a magnetic sorting system for islets microencapsulation purification (Fig. 6D). The cross-section of the main channel of the magnetic purification system is 1 mm × 1 mm, and the cross-section of the two channels bisected from the main channel is 750 μm × 750 μm. The system integrates a commercial neodymium magnet above the main channel near the bifurcation, so the magnetized microencapsulated islets move to top channel and empty capsules are evenly distributed between the two channels, resulting in the effective selective collection of magnetized samples in top outlet. Using this device, the graft volume of alginate microencapsulated islets and alginate-poly-L-lysine-alginate microcapsules was reduced by 77.5% and 78.6%, respectively. After subcutaneous implantation of purified alginate-poly-L-lysine-alginate microencapsulated pancreatic islets into Wistar rats with diabetes, blood glucose level returned to normal (< 200 mg/dL), and this effect lasted for nearly 17 weeks.

## Conclusion and outlook

Pancreatic islet organoids-on-a-chip as a new organoid culture and disease model platform is receiving extensive attention from researchers, perhaps this system can revolutionize the basic research and treatment of diabetes-related diseases. In this review, we recapitulate organoids-on-a-chip in the study of islet differentiation, maintenance and functional evaluation. We also present the applications of islet organoids-on-a-chip platform in disease modeling, drug screening and cell replacement therapy.

One advantage of islet organoids-on-a-chip is the ability to precisely control fluidic flow in channels, thereby enhancing the function of islets and maintaining its long-term viability [84]. For example, the morphology and function of islets can be maintained for as long as one month by optimizing the flow conditions of a microfluidic perfusion culture device [23]. Furthermore, hydrodynamic interactions between different gases, metabolites and cells inevitably affect islet viability and function, while well-designed architecture of organoids-on-a-chip using computational modeling allows precisely control of flow to eliminate these effects. By integrating analytical approaches (e.g., Raman microspectroscopy) with organoids-on-a-chip, the function of islet can be assessed fully automatically in real time. Another advantage of islet organoids-on-a-chip is its potential for wide use in biomedical research. Islet organoids-on-a-chip has more advantages than current animal models in drug screening owing to the poor correlation between animal experimental results and the data of clinical human trials, which is caused by key disease pathways of interspecies differences [85]. Finally, the application of islet organoids-on-a-chip platform to stem cell differentiation can potentially not only offer a sufficient source of high-quality islet organoids for islet transplantation, but also permit to study the molecular mechanisms of islet development to better prevent and treat pancreas-related diseases.

Although the field of islet organoids-on-a-chip is moving ahead at an impressive speed, it is still in early stage and there are many challenges to overcome before wide application. One key challenge is material to be used in rapid prototyping of chip, such as PDMS. It has been shown that PDMS assimilates hydrophobic molecules when be applied in cell culture substrates and drug screening devices, which may lead to a decrease in effective drug concentration and pharmacological activity [86, 87]. Polyurethane elastomer, an attractive alternative material, has similar properties to PDMS in optical transparency, flexibility and castability, but it is also resistant to absorption of small hydrophobic molecules [88]. What’s more, Polycarbonate as a scalable manufacturing material has been applied into the fabrication of microfluidic chip [47]. While continuous perfusion based on microfluidic systems generally supports long-term survival and real-time assessment of cells, air bubbles generated in the channels can hamper the control of chips and cause damage to cells, and these bubbles are usually difficult to eliminate. Fortunately, many researchers have developed a variety of methods to eliminate bubbles generated in microfluidics, such as by regulating the internal wettability [89], channel structure [90–92] and liquid flow rate [93]. Additional challenges include preventing microbial contamination, providing stable and continuous supply of nutrients to islet organoids, retrieving islet organoids from chips, and controlling interactions between cells and cells, and cells and ECM (extracellular matrix). With the development of material science and manufacturing technology, these problems may be solved by designing rational structures and utilizing advanced processing methods.

Opportunities often come with challenges. The development of the organoids-on-a-chip platform integrated with stem cell technology and materials science may allow us to overcome the abovementioned challenges. Achievements in the field of islet organoids-on-a-chip may provide a new theoretical basis for clinical treatment strategies for diabetes. We believe that the organoids-on-a-chip systems will eventually support the application of islet organoids in the new era of personalized and precise medicine in the near future.

## Data Availability

Not applicable.

## References

[1] Wang Y, Lo JF, Mendoza-Elias JE, Adewola AF, Harvat TA, Kinzer KP, Lee D, Qi M, Eddington DT, Oberholzer J (2010). Application of microfluidic technology to pancreatic islet research: first decade of endeavor. Bioanalysis.

[2] Lancaster MA, Knoblich JA (2014). Organogenesis in a dish: modeling development and disease using organoid technologies. Science.

[3] Clevers H (2016). Modeling development and disease with organoids. Cell.

[4] Park SE, Georgescu A, Huh D (2019). Organoids-on-a-chip. Science.

[5] Sun M, Liu A, Yang X, Gong J, Yu M, Yao X, Wang H, He Y (2021). 3D cell culture—can it be as popular as 2d cell culture?. Adv NanoBiomed Res.

[6] Yang K, Lee M, Jones PA, Liu SS, Zhou A, Xu J, Sreekanth V, Wu JLY, Vo L, Lee EA, Pop R, Lee Y, Wagner BK (2020). A 3D culture platform enables development of zinc-binding prodrugs for targeted proliferation of beta cells. Sci Adv.

[7] Tomasi RF, Sart S, Champetier T, Baroud CN (2020). Individual control and quantification of 3d spheroids in a high-density microfluidic droplet array. Cell Rep.

[8] McCauley HA, Wells JM (2017). Pluripotent stem cell-derived organoids: using principles of developmental biology to grow human tissues in a dish. Development.

[9] Jackson EL, Lu H (2016). Three-dimensional models for studying development and disease: moving on from organisms to organs-on-a-chip and organoids. Integr Biol (Camb).

[10] Harrison RG (1910). The outgrowth of the nerve fiber as a mode of protoplasmic kovement. TBS J Exp Zool.

[11] Ehrmann RL, Gey GO (1956). The growth of cells on a transparent gel of reconstituted rat-tail collagen. J Natl Cancer Inst.

[12] Mammoto T, Mammoto A, Ingber DE (2013). Mechanobiology and developmental control. Annu Rev Cell Dev Biol.

[13] Cerf ME (2011). Islet organogenesis, angiogenesis and innervation. Cell Biol Int.

[14] Li X, Brooks JC, Hu J, Ford KI, Easley CJ (2017). 3D-templated, fully automated microfluidic input/output multiplexer for endocrine tissue culture and secretion sampling. Lab Chip.

[15] Hirano K, Konagaya S, Turner A, Noda Y, Kitamura S, Kotera H, Iwata H (2017). Closed-channel culture system for efficient and reproducible differentiation of human pluripotent stem cells into islet cells. Biochem Biophys Res Commun.

[16] Kim JH, Park BG, Kim SK, Lee DH, Lee GG, Kim DH, Choi BO, Lee KB, Kim JH (2019). Nanotopographical regulation of pancreatic islet-like cluster formation from human pluripotent stem cells using a gradient-pattern chip. Acta Biomater.

[17] Essaouiba A, Okitsu T, Jellali R, Shinohara M, Danoy M, Tauran Y, Legallais C, Sakai Y, Leclerc E (2020). Microwell-based pancreas-on-chip model enhances genes expression and functionality of rat islets of Langerhans. Mol Cell Endocrinol.

[18] Sankar KS, Green BJ, Crocker AR, Verity JE, Altamentova SM, Rocheleau JV (2011). Culturing pancreatic islets in microfluidic flow enhances morphology of the associated endothelial cells. PLoS One.

[19] Jiang K, Chaimov D, Patel SN, Liang JP, Wiggins SC, Samojlik MM, Rubiano A, Simmons CS, Stabler CL (2019). 3-D physiomimetic extracellular matrix hydrogels provide a supportive microenvironment for rodent and human islet culture. Biomaterials.

[20] Liu H, Wang Y, Wang H, Zhao M, Tao T, Zhang X, Qin J (2020). A droplet microfluidic system to fabricate hybrid capsules enabling stem cell organoid engineering. Adv Sci (Weinh).

[21] Wang H, Liu H, Zhang X, Wang Y, Zhao M, Chen W, Qin J (2021). One-step generation of aqueous-droplet-filled hydrogel fibers as organoid carriers using an all-in-water microfluidic system. ACS Appl Mater Interfaces.

[22] Lee SH, Hong S, Song J, Cho B, Han EJ, Kondapavulur S, Kim D, Lee LP (2018). Microphysiological analysis platform of pancreatic islet beta-cell spheroids. Adv Healthc Mater.

[23] Jun Y, Lee J, Choi S, Yang JH, Sander M, Chung S, Lee S-H (2019). In vivo–mimicking microfluidic perfusion culture of pancreatic islet spheroids. Sci Adv.

[24] Tao T, Wang Y, Chen W, Li Z, Su W, Guo Y, Deng P, Qin J (2019). Engineering human islet organoids from iPSCs using an organ-on-chip platform. Lab Chip.

[25] Sneddon JB, Tang Q, Stock P, Bluestone JA, Roy S, Desai T, Hebrok M (2018). Stem cell therapies for treating diabetes: progress and remaining challenges. Cell Stem Cell.

[26] Ricordi C, Goldstein JS, Balamurugan AN, Szot GL, Kin T, Liu C, Czarniecki CW, Barbaro B, Bridges ND, Cano J, Clarke WR, Eggerman TL, Hunsicker LG, Kaufman DB, Khan A, Lafontant DE, Linetsky E, Luo X, Markmann JF, Ali Naji OK, Oberholzer J, Turgeon NA, Brandhorst D, Chen X, Friberg AS, Lei J, Wang LJ, Wilhelm JJ, Willits J, Zhang X, Hering BJ, Posselt AM, Stock PG, Shapiro AM (2016). National institutes of health–sponsored clinical islet transplantation consortium phase 3 trial: manufacture of a complex cellular product at eight processing facilities. Diabetes.

[27] Untereiner A, Abdo S, Bhattacharjee A, Gohil H, Pourasgari F, Ibeh N, Lai M, Batchuluun B, Wong A, Khuu N, Liu Y, Al Rijjal D, Winegarden N, Virtanen C, Orser BA, Cabrera O, Varga G, Rocheleau J, Dai FF, Wheeler MB (2019). GABA promotes beta-cell proliferation, but does not overcome impaired glucose homeostasis associated with diet-induced obesity. FASEB J.

[28] Wan X, Zinselmeyer BH, Zakharov PN, Vomund AN, Taniguchi R, Santambrogio L, Anderson MS, Lichti CF, Unanue ER (2018). Pancreatic islets communicate with lymphoid tissues via exocytosis of insulin peptides. Nature.

[29] Ishahak M, Birman L, Carbonero D, Hill J, Hernandez A, Rawal S, Agarwal A (2019). Integrated platform for operating and interrogating organs-on-chips. Anal Methods.

[30] Negou JT, Avila LA, Li X, Hagos TM, Easley CJ (2017). Automated microfluidic droplet-based sample chopper for detection of small fluorescence differences using lock-in analysis. Anal Chem.

[31] Perrier R, Pirog A, Jaffredo M, Gaitan J, Catargi B, Renaud S, Raoux M, Lang J (2018). Bioelectronic organ-based sensor for microfluidic real-time analysis of the demand in insulin. Biosens Bioelectron.

[32] Adewola AF, Lee D, Harvat T, Mohammed J, Eddington DT, Oberholzer J, Wang Y (2010). Microfluidic perifusion and imaging device for multi-parametric islet function assessment. Biomed Microdevices.

[33] Lee D, Wang Y, Mendoza-Elias JE, Adewola AF, Harvat TA, Kinzer K, Gutierrez D, Qi M, Eddington DT, Oberholzer J (2012). Dual microfluidic perifusion networks for concurrent islet perifusion and optical imaging. Biomed Microdevices.

[34] Lam AK, Silva PN, Altamentova SM, Rocheleau JV (2012). Quantitative imaging of electron transfer flavoprotein autofluorescence reveals the dynamics of lipid partitioning in living pancreatic islets. Integr Biol (Camb).

[35] Chen W, Lisowski M, Khalil G, Sweet IR, Shen AQ (2012). Microencapsulated 3-dimensional sensor for the measurement of oxygen in single isolated pancreatic islets. PLoS One.

[36] Nourmohammadzadeh M, Lo JF, Bochenek M, Mendoza-Elias JE, Wang Q, Li Z, Zeng L, Qi M, Eddington DT, Oberholzer J, Wang Y (2013). Microfluidic array with integrated oxygenation control for real-time live-cell imaging: effect of hypoxia on physiology of microencapsulated pancreatic islets. Anal Chem.

[37] Heileman K, Daoud J, Hasilo C, Gasparrini M, Paraskevas S, Tabrizian M (2015). Microfluidic platform for assessing pancreatic islet functionality through dielectric spectroscopy. Biomicrofluidics.

[38] Dishinger JF, Reid KR, Kennedy RT (2009). Quantitative monitoring of insulin secretion from single islets of langerhans in parallel on a microfluidic chip. Anal Chem.

[39] Yi L, Wang X, Dhumpa R, Schrell AM, Mukhitov N, Roper MG (2015). Integrated perfusion and separation systems for entrainment of insulin secretion from islets of Langerhans. Lab Chip.

[40] Godwin LA, Pilkerton ME, Deal KS, Wanders D, Judd RL, Easley CJ (2011). Passively operated microfluidic device for stimulation and secretion sampling of single pancreatic islets. Anal Chem.

[41] Zhang X, Grimley A, Bertram R, Roper MG (2010). Microfluidic system for generation of sinusoidal glucose waveforms for entrainment of islets of langerhans. Anal Chem.

[42] Lenguito G, Chaimov D, Weitz JR, Rodriguez-Diaz R, Rawal SA, Tamayo-Garcia A, Caicedo A, Stabler CL, Buchwald P, Agarwal A (2017). Resealable, optically accessible, PDMS-free fluidic platform for ex vivo interrogation of pancreatic islets. Lab Chip.

[43] Patel SN, Ishahak M, Chaimov D, Velraj A, LaShoto D, Hagan DW, Buchwald P, Phelps EA, Agarwal A, Stabler CL (2021). Organoid microphysiological system preserves pancreatic islet function within 3D matrix. Sci Adv..

[44] Misun PM, Yesildag B, Forschler F, Neelakandhan A, Rousset N, Biernath A, Hierlemann A, Frey O (2020). In vitro platform for studying human insulin release dynamics of single pancreatic islet microtissues at high resolution. Adv Biosyst.

[45] Zbinden A, Marzi J, Schlunder K, Probst C, Urbanczyk M, Black S, Brauchle EM, Layland SL, Kraushaar U, Duffy G, Schenke-Layland K, Loskill P (2020). Non-invasive marker-independent high content analysis of a microphysiological human pancreas-on-a-chip model. Matrix Biol..

[46] Li X, Hu J, Easley CJ (2018). Automated microfluidic droplet sampling with integrated, mix-and-read immunoassays to resolve endocrine tissue secretion dynamics. Lab Chip.

[47] Glieberman AL, Pope BD, Zimmerman JF, Liu Q, Ferrier JP, Kenty JHR, Schrell AM, Mukhitov N, Shores KL, Tepole AB, Melton DA, Roper MG, Parker KK (2019). Synchronized stimulation and continuous insulin sensing in a microfluidic human islet on a chip designed for scalable manufacturing. Lab Chip.

[48] Nourmohammadzadeh M, Xing Y, Lee JW, Bochenek MA, Mendoza-Elias JE, McGarrigle JJ, Marchese E, Chun-Chieh Y, Eddington DT, Oberholzer J, Wang Y (2016). A microfluidic array for real-time live-cell imaging of human and rodent pancreatic islets. Lab Chip.

[49] Silva PN, Green BJ, Altamentova SM, Rocheleau JV (2013). A microfluidic device designed to induce media flow throughout pancreatic islets while limiting shear-induced damage. Lab Chip.

[50] Wang X, Yi L, Roper MG (2016). Microfluidic device for the measurement of amino acid secretion dynamics from murine and human islets of langerhans. Anal Chem.

[51] Castiello FR, Tabrizian M (2018). Multiplex surface plasmon resonance imaging-based biosensor for human pancreatic islets hormones quantification. Anal Chem.

[52] Rorsman P, Braun M (2013). Regulation of insulin secretion in human pancreatic islets. Annu Rev Physiol.

[53] Becker MW, Simonovich JA, Phelps EA (2019). Engineered microenvironments and microdevices for modeling the pathophysiology of type 1 diabetes. Biomaterials.

[54] Dolensek J, Rupnik MS, Stozer A (2015). Structural similarities and differences between the human and the mouse pancreas. Islets.

[55] MacDonald MJ, Longacre MJ, Stoker SW, Kendrick M, Thonpho A, Brown LJ, Hasan NM, Jitrapakdee S, Fukao T, Hanson MS, Fernandez LA, Odorico J (2011). Differences between human and rodent pancreatic islets: low pyruvate carboxylase, atp citrate lyase, and pyruvate carboxylation and high glucose-stimulated acetoacetate in human pancreatic islets. J Biol Chem.

[56] Skelin Klemen M, Dolensek J, Slak Rupnik M, Stozer A (2017). The triggering pathway to insulin secretion: functional similarities and differences between the human and the mouse beta cells and their translational relevance. Islets.

[57] Zschaler J, Schlorke D, Arnhold J (2014). Differences in innate immune response between man and mouse. Crit Rev Immunol.

[58] Huh D, Matthews BD, Mammoto A, Montoya-Zavala M, Hsin HY, Ingber DE (2010). Reconstituting organ-level lung functions on a chip. Science.

[59] Materne EM, Tonevitsky AG, Marx U (2013). Chip-based liver equivalents for toxicity testing–organotypicalness versus cost-efficient high throughput. Lab Chip.

[60] Benam KH, Villenave R, Lucchesi C, Varone A, Hubeau C, Lee HH, Alves SE, Salmon M, Ferrante TC, Weaver JC, Bahinski A, Hamilton GA, Ingber DE (2016). Small airway-on-a-chip enables analysis of human lung inflammation and drug responses in vitro. Nat Methods.

[61] Lanng S (2001). Glucose intolerance in cystic fibrosis patients. Paediatr Respir Rev.

[62] Moran A, Dunitz J, Nathan B, Saeed A, Holme B, Thomas W (2009). Cystic fibrosis-related diabetes: current trends in prevalence, incidence, and mortality. Diabetes Care.

[63] Shik Mun K, Arora K, Huang Y, Yang F, Yarlagadda S, Ramananda Y, Abu-El-Haija M, Palermo JJ, Appakalai BN, Nathan JD, Naren AP (2019). Patient-derived pancreas-on-a-chip to model cystic fibrosis-related disorders. Nat Commun.

[64] Saltiel AR, Kahn CR (2001). Insulin signalling and the regulation of glucose and lipid metabolism. Nature.

[65] Leahy JL (2005). Pathogenesis of type 2 diabetes mellitus. Arch Med Res.

[66] Perry RJ, Samuel VT, Petersen KF, Shulman GI (2014). The role of hepatic lipids in hepatic insulin resistance and type 2 diabetes. Nature.

[67] Bauer S, Wennberg Huldt C, Kanebratt KP, Durieux I, Gunne D, Andersson S, Ewart L, Haynes WG, Maschmeyer I, Winter A, Ammala C, Marx U, Andersson TB (2017). Functional coupling of human pancreatic islets and liver spheroids on-a-chip: towards a novel human ex vivo type 2 diabetes model. Sci Rep.

[68] Tao T, Deng P, Wang Y, Zhang X, Guo Y, Chen W, Qin J (2022). Microengineered multi-organoid system from hipscs to recapitulate human liver-islet axis in normal and type 2 diabetes. Adv Sci (Weinh).

[69] Krebs MG, Sloane R, Priest L, Lancashire L, Hou JM, Greystoke A, Ward TH, Ferraldeschi R, Hughes A, Clack G, Ranson M, Dive C, Blackhall FH (2011). Evaluation and prognostic significance of circulating tumor cells in patients with non-small-cell lung cancer. J Clin Oncol.

[70] Mo SJ, Lee JH, Kye HG, Lee JM, Kim EJ, Geum D, Sun W, Chung BG (2020). A microfluidic gradient device for drug screening with human iPSC-derived motoneurons. Analyst.

[71] You X, Zhang X, Luo Y, Liu L, Zhao W, Lin B (2019). Establishment of 3D organ chip for multiplexed assessment of type 2 diabetes drugs. Prog Biochem Biophys.

[72] Nguyen DT, van Noort D, Jeong IK, Park S (2017). Endocrine system on chip for a diabetes treatment model. Biofabrication.

[73] Wassmer CH, Lebreton F, Bellofatto K, Bosco D, Berney T, Berishvili E (2020). Generation of insulin-secreting organoids: a step toward engineering and transplanting the bioartificial pancreas. Transpl Int.

[74] Papas KK, Avgoustiniatos ES, Suszynski TM (2016). Effect of oxygen supply on the size of implantable islet-containing encapsulation devices. Panminerva Med.

[75] Komatsu H, Cook C, Wang CH, Medrano L, Lin H, Kandeel F, Tai YC, Mullen Y (2017). Oxygen environment and islet size are the primary limiting factors of isolated pancreatic islet survival. PLoS One.

[76] Jun Y, Kang AR, Lee JS, Park SJ, Lee DY, Moon SH, Lee SH (2014). Microchip-based engineering of super-pancreatic islets supported by adipose-derived stem cells. Biomaterials.

[77] Hori T, Yamane K, Anazawa T, Kurosawa O, Iwata H (2019). Compact fluidic system for functional assessment of pancreatic islets. Biomed Microdevices.

[78] Naftanel MA, Harlan DM (2004). Pancreatic islet transplantation. PLoS Med.

[79] Buder B, Alexander M, Krishnan R, Chapman DW, Lakey JR (2013). Encapsulated islet transplantation: strategies and clinical trials. Immune Netw.

[80] Robles L, Storrs R, Lamb M, Alexander M, Lakey JR (2014). Current status of islet encapsulation. Cell Transplant.

[81] Robert R, Jean-Francois P, Francois AL, Michel L, Yves L, Jean-Pierre H (1999). Studies on small (< 350 µm) alginate-poly-L-lysine microcapsules. III. Biocompatibility of smaller versus standard microcapsules. J Biomed Mater Res..

[82] Zhang Y, Alexander M, Yang S, Bian Y, Botvinick E, Lakey JRT, Ozcan A (2018). High-throughput screening of encapsulated islets using wide-field lens-free on-chip imaging. ACS Photonics.

[83] Espona-Noguera A, Etxebarria-Elezgarai J, Saenz Del Burgo L, Canibano-Hernandez A, Gurruchaga H, Blanco FJ, Orive G, Hernandez RM, Benito-Lopez F, Ciriza J, Basabe-Desmonts L, Pedraz JL (2019). Type 1 diabetes mellitus reversal via implantation of magnetically purified microencapsulated pseudoislets. Int J Pharm..

[84] Bhatia SN, Ingber DE (2014). Microfluidic organs-on-chips. Nat Biotechnol.

[85] Esch EW, Bahinski A, Huh D (2015). Organs-on-chips at the frontiers of drug discovery. Nat Rev Drug Discov.

[86] van der Meer AD, van den Berg A (2012). Organs-on-chips: breaking the in vitro impasse. Integr Biol (Camb).

[87] Berthier E, Young EW, Beebe D (2012). Engineers are from PDMS-land, biologists are from polystyrenia. Lab Chip.

[88] Domansky K, Leslie DC, McKinney J, Fraser JP, Sliz JD, Hamkins-Indik T, Hamilton GA, Bahinski A, Ingber DE (2013). Clear castable polyurethane elastomer for fabrication of microfluidic devices. Lab Chip.

[89] Chen JD, Chen D, Xie Y, Chen X, Wang K, Cui DX, Du HX, Wang ZG (2015). Bubble generation and mechanism in polydimethylsiloxane based polymerase chain reaction chip. Applied Physics Letters.

[90] Sung JH, Shuler ML (2009). Prevention of air bubble formation in a microfluidic perfusion cell culture system using a microscale bubble trap. Biomed Microdevices.

[91] Huang C, Wippold JA, Stratis-Cullum D, Han A (2020). Eliminating air bubble in microfluidic systems utilizing integrated in-line sloped microstructures. Biomed Microdevices.

[92] Stucki JD, Guenat OT (2015). A microfluidic bubble trap and oscillator. Lab Chip.

[93] He X, Wang BS, Meng JX, Zhang SD, Wang ST (2021). How to prevent bubbles in microfluidic channels. Langmuir.

